# Evolutionary and social consequences of introgression of nontransgenic herbicide resistance from rice to weedy rice in Brazil

**DOI:** 10.1111/eva.12387

**Published:** 2016-06-25

**Authors:** Aldo Merotto, Ives C. G. R. Goulart, Anderson L. Nunes, Augusto Kalsing, Catarine Markus, Valmir G. Menezes, Alcido E. Wander

**Affiliations:** ^1^ Federal University of Rio Grande do Sul—UFRGSPorto AlegreRSBrazil; ^2^ Brasilian Agriculture Research Corporation—EMBRAPA ForestryColomboPRBrazil; ^3^ Federal Institute of Rio Grande do Sul—IFRSSertãoRSBrazil; ^4^ Dow AgroSciences Ind. LtdaSão PauloSPBrazil; ^5^ Institute Rio‐Grandense of Rice—IRGACachoerinhaRSBrazil; ^6^ Brasilian Agriculture Research Corporation—EMBRAPA Rice and BeansSanto Antônio de GoiasGOBrazil

**Keywords:** acetolactate synthase, Clearfield^TM^, fitness, gene flow, herbicide resistance, imazethapyr, outcrossing, red rice

## Abstract

Several studies have expressed concerns about the effects of gene flow from transgenic herbicide‐resistant crops to their wild relatives, but no major problems have been observed. This review describes a case study in which what has been feared in transgenics regarding gene flow has actually changed biodiversity and people's lives. Nontransgenic imidazolinone‐resistant rice (IMI‐rice) cultivars increased the rice grain yield by 50% in southern Brazil. This increase was beneficial for life quality of the farmers and also improved the regional economy. However, weedy rice resistant to imidazolinone herbicides started to evolve three years after the first use of IMI‐rice cultivars. Population genetic studies indicate that the herbicide‐resistant weedy rice was mainly originated from gene flow from resistant cultivars and distributed by seed migration. The problems related with herbicide‐resistant weedy rice increased the production costs of rice that forced farmers to sell or rent their land. Gene flow from cultivated rice to weedy rice has proven to be a large agricultural, economic, and social constraint in the use of herbicide‐resistant technologies in rice. This problem must be taken into account for the development of new transgenic or nontransgenic rice technologies.

## Introduction

Gene flow is a major source of genetic variability in plants, animals, and humans and, together with natural selection and genetic drift, maintains the evolution of life (Harlan 1975). The gene flow from crops to related species has been reported in various genera such as *Brassica*,* Helianthus*,* Oryza*,* Raphanus*,* Sorghum,* and *Zea* (Mallory‐Smith and Zapiola 2008; Ellstrand et al. 2010). Major concerns about gene flow have been expressed in transgenic crops, such as the herbicide‐resistant *Brassica napus* (Devos et al. 2012; Liu et al. 2013) and *Agrostis stolonifera* (Zapiola et al. 2008; Zapiola and Mallory‐Smith 2012). These species have wild types that could facilitate the escape of the herbicide‐tolerant trait to agricultural or semi‐natural habitats. However, transgenic crops have not caused substantial environmental or economic losses associated with gene flow (Green 2012). On the other hand, the gene flow from nontransgenic imidazolinone‐resistant rice (IMI‐rice) cultivars to weedy rice has actually changed the lives of farmers and people.

The gene flow that occurs in rice is one of the most significant examples of this process because rice is an important staple food and have a large number of species or wild types, including weedy (red) rice, that are able to hybridize with cultivated rice. Weedy rice is described as genetically diverse populations of cultivated rice (*Oryza sativa* L.) that has adapted to coevolve with this crop (Gealy et al. 2015). Weedy rice is one of the most significant problems in the majority of cultivated rice fields worldwide (Sudianto et al. 2013; Ziska et al. 2015). Weedy rice severely decreases the grain yield and profitability of rice, increases production costs, and depreciates the harvested product (Noldin et al. 2004). Weedy rice could reduce rice grain yield by 90% in heavily infested areas (Diarra et al. 1985). In the United States, the average loss due to red rice infestation is nearly $274.00 ha^−1^ (Burgos et al. 2008).

Contrary to common belief, the red color of the grain is not the major problem of weedy rice. In fact, several biotypes do not have the red pericarp (Arrieta‐Espinoza et al. 2005), and the red‐colored grains of rice are edible and consumed as a special food (Finocchiaro et al. 2007). The major harm of the weediness and persistence of weedy rice is associated with high seed shattering (Nunes et al. 2015), high seed dormancy (Schwanke et al. 2008), and high competitive capacity due to its taller growth and higher tillering compared to cultivated rice (Fleck et al. 2008). Weedy rice and cultivated rice belong to the same species, *Oryza sativa* (Gross et al. 2010), therefore, selective herbicides commonly used for weed control cannot be used to kill contaminating weedy rice. In the 1990s, an imidazolinone‐resistant rice (IMI‐rice) line named 93‐AS‐3510 was obtained through ethyl methanesulfonate‐induced mutagenesis (Croughan 1998). This line was used to develop the IMI‐rice cultivars CL 121 and CL 141 in the United States and IRGA 422 CL in Brazil (Roso et al. 2010b; Sudianto et al. 2013). A similar approach was used to develop the line PWC16 (Wenefrida et al. 2004), which was further used to create the IMI‐rice CL 161 in the United States and several hybrid cultivars in Brazil (Roso et al. 2010b). In addition, the IMI‐rice PUITÁ INTA CL was also developed in Argentina through artificial mutation (Livore 2003). Based on these primary cultivars, several others were developed in the United States, Brazil, and other regions where IMI‐rice is been used. The point mutations in the ALS (acetolactate synthase, also known as acetohydroxy acid synthase or AHAS) gene that confer resistance to the IMI‐rice lines 93‐AS‐3510, PWC13, and Puitá INTA CL were Gly654Glu, Ser653Asn, and Ala122Thr, respectively (Roso et al. 2010b), which result in different levels of resistance to imidazolinone herbicides (Avila et al. 2005).

**Figure 1:**
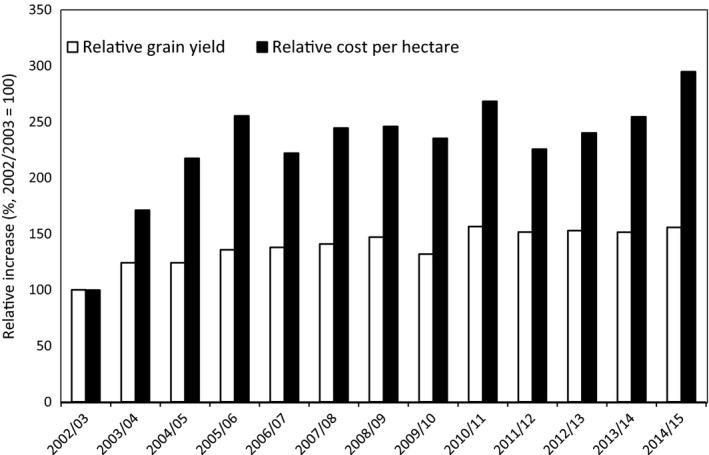
Progress of grain yields and per hectare costs of production of irrigate rice in Rio Grande do Sul state (Brazil), 2002/03 to 2014/15. Source: Adapted from CONAB (2015).

The introduction of IMI‐rice facilitated the selective control of weedy rice, which, together with other management practices, increased the rice grain yield in southern Brazil by approximately 2500 kg ha^−1^, an increase of 50% (Fig. 1). This technology had a significant economic and social impact in the southern part of the state of Rio Grande do Sul, where 1.1 million ha are cultivated and the rice business is the main source of income in several cities. The rapid increase in grain yield and revenue raised farmers' profitability and changed the economic and social characteristics of many rice‐producing regions. The significant benefits of IMI‐rice indicate that it is one of the most important technologies for the rice crop since the introduction of dwarf rice varieties in the 1970s. However, several biotypes of weedy rice resistant to ALS‐inhibiting herbicides were identified in rice paddy fields three growing seasons after the introduction of IMI‐rice to Brazil (Menezes et al. 2009; Roso et al. 2010b). Similar problems were found in the United States (Shivrain et al. 2007), Italy (Andres et al. 2014) and Greece (Kaloumenos et al. 2013). This problem is challenging the use of IMI‐rice because heavy infestations of weedy rice are still present in several fields similarly to the situation that occurred before the introduction of IMI‐rice technology.

Nowadays, several transgenic rice lines resistant to several herbicides (Ryffel 2014; Chhapekar et al. 2015; Tian et al. 2015) and other nontransgenic rice varieties resistant to ACCase‐inhibiting herbicides are being developed (Harden et al. 2014). The problems related to the consequences of gene flow of IMI‐rice should be considered for the development of these new transgenic and nontransgenic cultivars to avoid the occurrence of herbicide resistance in weedy rice. The southern region of Brazil is, nowadays, one of the largest areas worldwide where IMI‐rice cultivars are grown. The presence of herbicide‐resistant weedy rice, together with the characteristics of the different growing regions, makes this an appropriate case study for evaluating the impact of gene flow in a large cropping system. The aims of this review are to analyze the evolution of herbicide resistance in weedy rice and to discuss the economic and social impact of nontransgenic imidazolinone herbicide‐resistant rice cultivars in southern Brazil.

## Evolutionary consequences of gene flow to weedy rice

Rice is primarily an inbreeder, although several studies have found that the outcrossing rate ranges from 0.01% to 52% (Langevin et al. 1990; Gealy et al. 2003). Therefore, large gene introgression between cultivated rice and weedy rice can be expected. However, the evolution of the herbicide‐resistant trait in this crop–weed system is likely to occur even faster in comparison with other traits, such as insect resistance or stress tolerance. In the case of herbicide resistance evolution, most of the susceptible plants are consistently eliminated by the herbicide application, leaving the resistant plants as the primarily source of pollen. Moreover, after hybridization, hybrid vigor can result in a weedy rice population that is more competitive than its original relatives. This may result in higher economic losses in rice crop cultivation (Shivrain et al. 2009). Different from the United States where IMI‐rice is also cultivated in a large area and where a single ALS mutation is used as the predominant source of resistance, in Brazil, three different ALS mutations were used to develop the IMI‐rice varieties (Roso et al. 2010b). Therefore, the different consequences of these mutations at molecular level and the implications regarding herbicide use and crop management in this region resulted in several particularities in the evolution of herbicide resistance in weedy rice in comparison with similar processes that occurred elsewhere (Kaloumenos et al. 2013; Andres et al. 2014; Burgos et al. 2014).

### Identification of resistant plants and distribution of ALS‐resistant alleles

**Figure 2:**
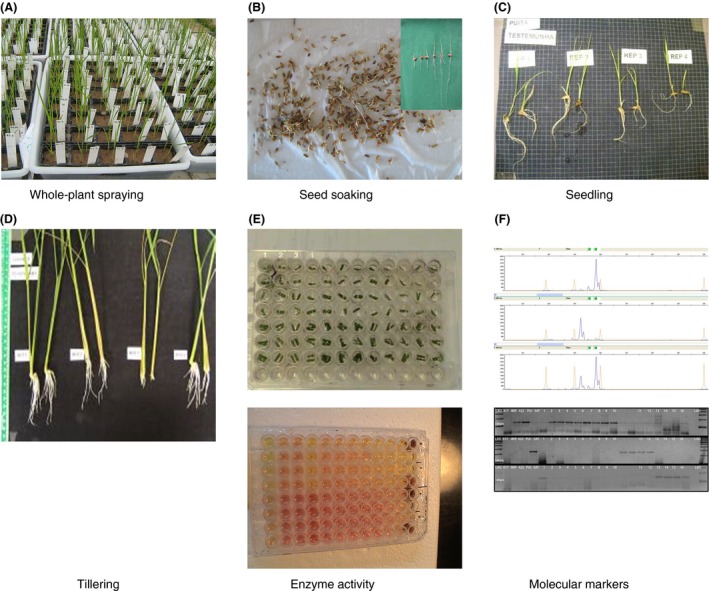
Illustrations of the diagnostic methods for herbicide resistance identification using whole‐plant herbicide spraying (A), seed soaking (B), seedling floating (C), tillering floating (D), enzyme activity (E), and molecular markers (F).

Herbicide resistance evolution in weedy rice in southern Brazil occurred differently than in other weed species where the resistance was initially suspected in a few plants or biotypes. The suspicion of resistance started in a large number of fields where a large number of weedy rice escapees of the imidazolinone herbicide application were found just three years after the beginning of the use of the IMI‐rice cultivars (Menezes et al. 2009). Therefore, methods to identify the weedy rice plants resistant to imidazolinone herbicides were developed based on bioassays in seeds, seedlings, and tillers, and SNP (single‐nucleotide polymorphism) molecular markers especially designed for the cultivated rice genotypes and mutations of the ALS gene used in southern Brazil (Roso et al. 2010a). These methods can identify the resistance in all stages of plant development (Fig. 2). Rapid identification of resistant specimens is critical for establishing correct decision‐making measures to prevent the distribution of resistant individuals. Advances in the use of methods for identification of herbicide resistance in weeds for the correct control of this problem should be performed in a similar way to those used in other areas related to the use of antibiotics (Trivedi and Rosenberg 2013) and the control of vector insects associated with human diseases (Perea et al. 2009). These advances depend on the adjustment of the herbicide‐resistant methods and on the motivation and training of the agricultural extension services and farmers.

The broad spread of imidazolinone‐resistant weedy rice plants in several regions of southern Brazil raised questions about the distribution of the different ALS alleles present in the IMI‐rice cultivars. An SNP molecular markers assay developed to identify the three mutations present in all IMI‐rice cultivars was performed in 481 plants from 38 populations collected as individuals that escaped from the imidazolinone herbicides in rice paddy fields (Roso et al. 2010b). The Gly654Glu mutation was found in 100% and 90.9% of the populations in the 2006/2007 and 2007/2008 seasons, respectively. The mutations Ser653Asn and Ala122Thr were also present either alone or as double or triple mutations in some plants. The results indicate that resistance to imidazolinone herbicides was mostly caused by the Gly654Glu mutation. This is the same mutation as that of the most used IMI‐rice cultivar at the time of evaluation, suggesting that gene flow was the origin of the herbicide resistance in weedy rice.

### Identification of the origin of herbicide resistance in weedy rice

Although the occurrence of gene flow between cultivated and weedy rice is likely the origin of the imidazolinone herbicide resistance, this also could be originated from independent evolution of a spontaneous mutation in the ALS gene (Delye et al. 2009). A paternity exclusion analysis (van Treuren et al. 2006) using SSR (simple sequenced repeat) markers and the SNP assay described earlier was performed to identify hybrids of the IMI‐rice cultivars (Goulart et al. 2011, 2012b). The results indicated that, in 98.9% of the weedy rice plants, the resistance to imidazolinone herbicides was caused by gene flow and that, in only 1.1% of the individuals, the resistance was caused by independent evolution (Goulart et al. 2012b). The theoretical rate of independent evolution, calculated based on gene flow rate, plant density, seed production, initial mutation frequency, and fitness penalty, was 2.9%. The difference between the obtained and calculated frequency of independent evolution as the cause of the herbicide resistance could be related to the compatibility between the IMI‐rice cultivars and weedy rice (Xia et al. 2011).

### Gene flow between weedy rice and hybrid and inbred rice cultivars

Several approaches regarding plant distribution have been used for the evaluation of gene flow in plants. Among these approaches is the allocation of donor plants in an area of 1 m² (Noldin et al. 2002) or 78 m² (Shivrain et al. 2007), in plots sown in different proportions (Olguin et al. 2009) and in greenhouses where the recipient plants are surrounded by donor plants (Merotto et al. 2009). However, the large presence of isolated plants broadly distributed in rice fields raised the question of the magnitude of gene flow in this scenario. Therefore, a field study was performed based on a single pollen‐donor plant in an encircled population combination technique with IMI‐rice cultivars carrying all the different mutations as pollen donors and a rice cultivar and a weedy rice ecotype as pollen receptors (Goulart et al. 2015). The use of a single donor plant in the center of an area of recipient plants favors the interpretation regarding situations where the resistant individuals may be derived from seed contaminants or where the resistance was originated from a spontaneous mutation in the ALS gene. In both situations, the resistant plant will have a low frequency within the population and the single donor plant approach simulated these scenarios.

The combinations of pollen‐donor and pollen‐receptor plants and the necessary sample size resulted in the evaluation of approximately one million seeds (Goulart et al. 2015), which were evaluated through the seed soaking, whole‐plant herbicide spraying, and SNP molecular markers as described above (Fig. 2). The outcrossing rate from the pollen receptor weedy rice biotype (0.0344%) was superior to that of the rice cultivar IRGA 417 (0.0142%). Some features, such as the protrusion of the stigma, the pistil length, the opening size of the anthers, and the number of grains deposited on the stigma are highly correlated with cross‐fertilization in rice (Matsui and Kagata 2003). These characteristics may be more prevalent in weedy rice than in cultivated rice, which would favor the occurrence of outcrossing and gene flow in weedy rice.

There was no significant variation in the pollen‐donor plants. The IMI‐rice inbred cultivars IRGA 422 CL and PUITÁ INTA CL, the hybrid SATOR CL, and the herbicide‐resistant weedy rice ecotype showed a similar outcrossing rate of 0.0243%. It was expected that the hybrid cultivars and weedy rice would have a higher pollen‐donor outcrossing rate than the other cultivars (Gealy et al. 2003). The obtained result may be related to the low outcrossing rate that was found in this study. Therefore, the amount of provided pollen may have been more than the necessary amount for fertilizing the pollen‐receptor plants, which resulted in equal outcrossing rates among these genotypes. In studies that were also performed under field conditions in the United States, the outcrossing rate for the pollen donor was higher in the hybrid cultivar CLXL8 (1.26%) than in the inbred cultivar CL 161 (0.21%) (Shivrain et al. 2009). It must be noted that, in this study, the outcrossing rates were 10 to 60 times higher than those found in the present evaluation and in other studies (Messeguer et al. 2004; Shivrain et al. 2007).

### Fitness effect associated with herbicide resistance in weedy rice

**Figure 3:**
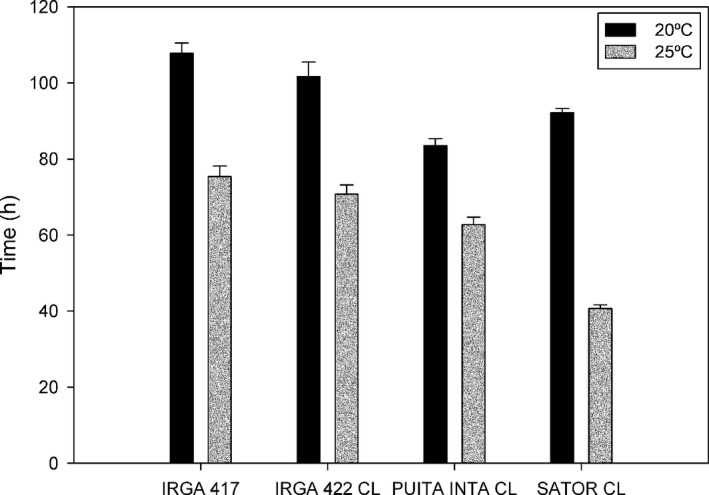
Time to germination reach 50% (GR 50%) based on the logistic model at temperatures of 20 and 25°C of rice cultivars carrying different ALS alleles. Source: Adapted from Goulart et al. 2012a .

One of the main risks of gene flow is the change in the fitness of the hybrid plants by incorporating beneficial alleles and increasing hybrid vigor (Ellstrand 2009; Olguin et al. 2009; Ellstrand et al. 2010). Several physiological processes can result in alterations of plant growth and development, resulting in changes in seed production. The different gene mutations associated with ALS‐inhibiting herbicide resistance can decrease the feedback regulation of the branched chain amino acids biosynthesis, which results in seeds with higher amino acids content (Dyer et al. 1993). These seeds could have a faster germination, mainly in temperatures lower than the optimum for germination, in comparison with seeds from the nonmutated plants (Yu et al. 2010). Studies were conducted to evaluate whether changes in the pattern of IMI‐rice cultivar germination are related to the consequences of different mutations of the ALS gene or whether they are due to environmental effects (Goulart et al. 2012a). The existence of rice cultivars with three different ALS gene alleles and the close paternity of these cultivars facilitate the identification of the fitness effect associated with these mutations. The IMI‐rice cultivar Puitá INTA CL carrying the ALS mutation Ala122Thr germinated faster than the susceptible rice cultivar at 20°C but not at 25 or 30°C (Fig. 3). Therefore, weedy rice individuals carrying this mutation could have a significant advantage due to the faster germination, mainly in early season seeding. In this situation, the earlier growing plants will be more competitive and are less likely to be eliminated by the herbicide application, contributing to a larger seed set in comparison with the susceptible plants. This process can contribute to increase the problem of the occurrence of herbicide resistance in weedy rice.

The variation of plant development and seed production associated with the different ALS gene mutations was evaluated based on artificial hybrids obtained between IMI‐rice cultivars and weedy rice (Nunes et al. 2010). The F1 and F2 hybrids were evaluated in comparison to their parents in environments with low and high nutrient availability. The results indicated a similar plant height between hybrids and weedy rice. However, hybrids resulting from the Puitá INTA CL and Sator CL cultivars produced more seeds than their weedy rice parents in both nutrient environments. Another important trait in the evolution of weedy rice in agricultural systems is seed shattering (Thurber et al. 2010). The hybrids obtained from all evaluated cultivars showed less shattering than weedy rice (Nunes et al. 2010). The fitness effect related with the faster germination can be associated with the consequence of the herbicide‐resistant gene as described above. However, the effects on seed production and seed shattering are not specifically related with the different ALS gene mutations and are associated with the broad genotype interaction between pollen donor and receptor. However, the consequences related to the variation of adaptation in hybrids of herbicide‐resistant plants are less important in comparison with other plant traits because the herbicide effect will eliminate the susceptible individuals.

### Population structure of weedy rice and its relationship with the wild *Oryza* species

**Figure 4:**
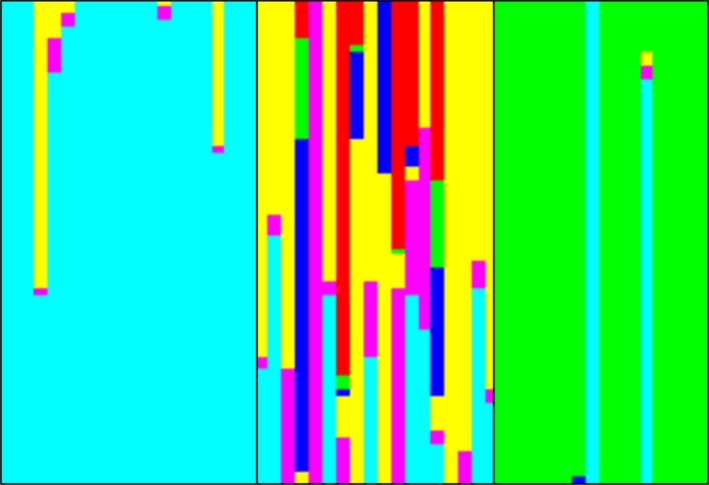
Illustration of the structure analysis of weedy rice populations. Each population comprises 20 plants represented by a vertical bar assigned to six potential genetic backgrounds (six different colors). The analysis was performed with the software structure as described in Goulart et al. (2014).

The rapid evolution of weedy rice resistance to imidazolinone can be explained not only by the gene flow of pollen between plants and by the adaptive variation but also by the weedy rice seed movement. The predominance of selfing in the genus *Oryza* determines that gene flow is commonly restricted, and high genetic diversity among ecologically distinct populations is expected (Xia et al. 2011; Jiang et al. 2012). In addition, the possibility of introgression between weedy rice with the wild *Oryza* species is a concern related to the consequences of gene flow to the native species (Lu and Snow 2005). A study analyzed the relationship of weedy rice populations from southern Brazil with rice cultivars and the wild *Oryza* species (Goulart et al. 2014). This study identified the occurrence of seed migration as the distribution mechanism of herbicide resistance in weedy rice. The genetic variation among and within weedy rice populations was 26 and 74%, respectively. The F_ST_ value of 0.26 found in this study is lower than that of similar analyses, ranging from 0.47 in a study that compared *japonica* and *indica‐*type weedy rice (Chung and Park 2010) to 0.76 obtained by comparing straw and blackhulled weedy rice populations (Londo and Schaal 2007). The obtained results indicate that seed migration of weedy rice as a contaminant of cultivated rice seed lots was occurring and was an important source of the origin of herbicide resistance in paddy fields where susceptible weedy rice was present. Other evidence found was the lack of correlation (*R*² = 0.06) between the genetic and geographic distances of the assessed populations (Goulart et al. 2014), indicating a large distance seed migration. In this study, for example, a resistant population with a highly homogeneous genetic background was identified, indicating the absence of migration (Pop 1), a resistant population with a high degree of multiple introductions (Pop 2) and a predominantly susceptible population with two resistant plants that were certainly recently introduced (Pop 3) (Fig. 4).

The majority of the weedy rice genetic background was associated with the new *indica*‐type cultivars used since the late 1970s, as opposed to the *japonica*‐type cultivars used in earlier years (Goulart et al. 2014). Weedy rice was largely present in the period when the *japonica*‐type cultivars were used in southern Brazil. Therefore, gene flow between weedy and cultivated rice during the last, approximately, 35 years, replaced the *japonica* by the *indica‐*type genetic background in weedy rice. Weedy rice clustered separately from the South American native *O. glumaepatula* and also from *O. rufipogon, O. longistaminata,* and *O. glaberrima,* indicating a low likelihood of introgression between weedy and cultivated rice with these AA genome wild *Oryza* species (Goulart et al. 2014).

The large genetic variability found within weedy rice populations in southern Brazil is related to gene flow among rice fields caused by seed migration. This, in turn, is related to the farmer's low use of certified seeds, which occurred mainly when using the new cultivars that had a high seed cost. This is a problem that challenges the use of the IMI‐rice cultivars as demonstrated in this study and that should be considered for the development of new transgenic or nontransgenic rice cultivars.

## Economic and social consequences of IMI‐rice in southern Brazil

The IMI‐rice history in southern Brazil is a classic case of the *Tragedy of the Commons*. Hardin (1968) defined the *Tragedy of the Commons* as the consequences of the use of a process by few individuals who take quick and maximum advantage in detriment of sustainable use for long‐term collective benefits. It is important to emphasize that, before IMI‐rice availability, most paddy fields were highly infested with weedy rice, and some areas were abandoned. In addition, the region's average rice grain yield was stacked at 5500 kg ha^−1^, despite the 12 000 kg ha^−1^ obtained in areas not infested by weedy rice (Merotto et al. 2013). The adoption of the IMI‐rice technology was very fast, even with the initial high cost of cultivated rice seeds and herbicides. The IMI‐rice use occurred together with the implementation of the extension programs called Projeto10, CFC, and Marca that targeted correct implementation of crop management practices. In the beginning, the use of IMI‐rice benefited all the rice farmers. However, the high frequency of weedy rice resistant to the imidazolinone herbicides, caused mainly by gene flow from the cultivated rice and broadly distributed by cultivated rice seed contaminated with weedy rice seeds, challenged the continuity of the use of the IMI‐rice technology between 2008 and 2010. In this period, production costs were high (Fig. 1), and the previous benefits of the continuous increase of the grain yield did not occur any more. After this period, variability among farmers started to occur and the high weedy rice infestation showed several consequences. The farmers that had low grain yields faced low profitability, and several of them went out of business, selling or renting their land. While average yield increased by 56% between 2002/2003 and 2014/2015, the production costs per hectare increased by 201% in the same period (Fig. 1), challenging the profitability of rice crops in this region.

The high price of soybeans that occurred globally in the 2010s increased the possibility of using this crop in rotation with rice, mainly in highly herbicide‐resistant infested areas. Although the soybean grain yields in these areas were limited by drought or flooding, depending on the rain patterns and soil characteristics, the high soybean price resulted in reasonable profitability in most fields. However, soil type, availability of machinery, and farm size limited the possibility of crop rotation with soybean in several rice areas. In addition, the use of the IMI‐rice cultivar Puitá INTA CL had increased since approximately 2010, providing better weedy rice control. This cultivar carried the mutation Ala122Thr that has a high level of herbicide resistance in comparison with the cultivar IRGA 422 CL. The larger herbicide tolerance of Puitá INTA CL allowed the use of high herbicide doses applied at pre‐ and postemergence as opposed to the postemergence with the cultivar IRGA 422 CL. In addition, some farmers became aware of the problem of cultivated rice seeds contaminated with weedy rice seeds, which led to an increase in the use of certified seeds. The result of the combination of crop rotation, better crop management, herbicide use, and noncontaminated seeds, among other measures, provided adequate weedy rice control, and some farmers were able to obtain high grain yields and profitability. However, soybean areas used in rotation with rice are treated continually with glyphosate, which creates a highly dangerous scenario for the evolution of weedy rice resistant to this herbicide.

The social effects of IMI‐rice were present at the farm and state level. The high profitability of rice had a positive impact on the quality of life of the farmers. Due to the increase of business activity at the regional level, this was also transferred to people who were not directly related to the rice crop industry. State rice tax revenues increased by 109%, which distributed the benefits to the whole population. However, the evolution of herbicide resistance in weedy rice decreased these benefits and resulted in changes in the whole rice farm structure, creating an impact on the whole region of southern Brazil where this crop is cultivated.

## Conclusions and future direction

Gene flow is a major issue, and it is challenging the use of IMI‐rice in southern Brazil. The benefits of IMI‐rice cultivars in this area overcame the weed control practices and increased the rice grain yield by approximately 50%. In the first five years of using IMI‐rice technology, the rice profitability for all farmers increased, resulting in a high social and economic impact at the regional and state level. However, gene flow from IMI‐rice cultivars to weedy rice resulted in the broad occurrence of imidazolinone herbicide‐resistant weedy rice just three years after the introduction of the IMI‐rice technology. Independent evolution of the herbicide resistance occurred in only 1.1% of the weedy rice plants, indicating that gene flow was the major cause of resistance. The population structure analysis indicates that the resistance was rapidly spread by the use of uncertified seeds of cultivated rice that were contaminated with weedy rice seeds.

Weedy rice resistance to IMI‐herbicides is an example of how gene flow affects the lives of farmers and people. The problems associated with the herbicide resistance in weedy rice generate a breakdown in the broad benefits of IMI‐rice. The increase of production costs, driven by the initially high rice profitability, outweighed the increase of the rice grain yield. Several consequences occurred because of the imidazolinone resistance in weedy rice and the increase of production costs. Several rice growers left the business by selling or renting their lands, which led to increase the average farm size. However, IMI‐rice cultivars are still used in approximately 50% of the rice areas in southern Brazil. Nowadays, the use of IMI‐rice technology is based on crop rotation with soybean, IMI‐rice cultivars with greater herbicide tolerance that provides better weedy rice control, use of certified cultivated rice seeds, alternation between wet seeding and dry drill‐seeded establishment systems, and hand‐pulling of weedy rice escapees. Different adjustments of these practices are being used in small and large farms to provide the adequate crop and weed management of IMI‐rice.

IMI‐rice cultivars are starting to be used in several rice growing regions of Asia and South and Central America. The consequences related to the gene flow to weedy rice that occurred in the major areas where IMI‐rice has been used in southern Brazil, as described in this review, and in the United States (Gealy et al. 2003; Sudianto et al. 2013; Burgos et al. 2014) and Europe (Kaloumenos et al. 2013; Andres et al. 2014) should be considered to avoid or minimize the problems associated with this technology. The ALS gene mutations Ala122Thr and Ser653Asn that confer high level of resistance should be used for developing local IMI‐rice cultivars. The rice cultivars with these mutations tolerate the herbicide dose necessary to control weedy rice. In addition, the herbicide use program should not consider the application only at postemergence, even though it is considered more practical and economical. The weedy rice plants that escape this application will not be controlled and will produce seeds. Therefore, the herbicide program should consider imidazolinone herbicide use at pre‐ and postemergence. The other practices related to the use of certified seeds, crop rotation and the efficiency of the herbicide application depend on effective farm extension services and the attitude of the farmers. The diagnosis of herbicide resistance in weedy rice (Roso et al. 2010a) is an innovation that should be used in the areas where IMI‐rice is being used or in new regions where this technology is in development.

The consequences of gene flow between cultivated rice and weedy rice are a strong limitation for the development of transgenic rice resistant to herbicides. This problem is also important for the nontransgenic herbicide‐resistant rice, but this technology is not regulated in relation to the gene flow consequences. Several naturally occurring or genetically engineered processes have been proposed to contain or mitigate gene flow. These processes could be applied to the cultivated–weedy rice system. The containment approach is based on maternal inheritance, male sterility, apomixes, and cleistogamy, aiming to prevent gene movement from the crop to the co‐specific weeds (Yoshida et al. 2007; Lin et al. 2008; Shi et al. 2008). The mitigation strategy consists of the incorporation of genes, such as those resulting in dwarfism, low seed shattering, and susceptibility to herbicides, together with the gene of interest, to decrease the establishment of the transgene in the population (Al‐Ahmad et al. 2004; Rose et al. 2009).

Although most containment or mitigation technologies do not completely eliminate the gene flow consequences (Gressel 2015), they decrease gene introgression and the establishment of new plants, as opposed to herbicide‐resistant cultivars where gene flow occurs naturally. The regulation of most of these processes is complex mainly in the weedy types of the cospecific crops. For example, the seed shattering regulation in weedy rice is under the control of multiple genes (Nunes et al. 2014, 2015; Subudhi et al. 2015) as opposed to the presence of a major gene in cultivated rice of the *indica* (Li et al. 2006) or *japonica* type (Konishi et al. 2006). Therefore, the development of the biotechnological‐based strategies designed for reducing gene flow between cultivated rice and weedy rice should be taken into account for the multiple regulation of the trait involved in the gene flow containment or mitigation. These complex solutions and its own drawbacks indicate that gene flow to weedy rice will continue to be a challenge for the development of transgenic or nontransgenic biotechnological processes and for the weed and crop management in rice.
